# Functional and homeostatic defects of regulatory T cells in patients with coronary artery disease

**DOI:** 10.1111/joim.12398

**Published:** 2015-08-11

**Authors:** L. Hasib, A. K. Lundberg, H. Zachrisson, J. Ernerudh, L. Jonasson

**Affiliations:** ^1^ Division of Cardiovascular MedicineDepartment of Medical and Health SciencesLinköping UniversityLinköpingSweden; ^2^ Department of Clinical PhysiologyLinköping UniversityLinköpingSweden; ^3^ Division of Clinical ImmunologyDepartment of Clinical and Experimental MedicineLinköping UniversityLinköpingSweden

**Keywords:** acute coronary syndrome, coronary artery disease, immune homeostasis, inflammation, regulatory T cell

## Abstract

**Objective:**

Regulatory T cells (Tregs) are considered atheroprotective, and low levels have been associated with the acute coronary syndrome (ACS), particularly non‐ST elevation (NSTE)‐ACS. However, the functional properties as well as homeostasis of Tregs are mainly unknown in coronary artery disease (CAD). Here, we investigated the composition and functional properties of naïve (n) and memory (m)Tregs in patients with NSTE‐ACS and in patients 6–12 months post‐ACS.

**Methods:**

Based on the expression of CD25, FOXP3, CD127, CD45RA, CD39 and CTLA‐4, Treg subsets were defined by flow cytometry in whole blood or isolated CD4^+^ T cells. The functional properties of nTregs and mTregs were examined in terms of proliferative capacity and modulation of cytokine secretion. To understand the potential consequences of Treg defects, we also investigated correlations with lipopolysaccharide (LPS)‐induced cytokine secretion and ultrasound‐defined carotid atherosclerosis.

**Results:**

Both NSTE‐ACS and post‐ACS patients exhibited reduced levels of nTregs (*P *<* *0.001) compared with healthy control subjects, but without compensatory increases in mTregs. Both nTregs and mTregs from patients showed significantly lower replicative rates and impaired capacity to modulate T‐cell proliferation and secretion of interferon‐gamma and IL‐10. The Treg defect was also associated with LPS‐induced cytokine secretion and increased burden of carotid atherosclerosis.

**Conclusion:**

Our results demonstrate a functional and homeostatic Treg defect in patients with NSTE‐ACS and also in stabilized patients 6–12 months after ACS. Moreover, this defect was associated with a subclinical proinflammatory and atherogenic state. We believe that the failure to preserve Treg function and homeostasis reflects a need for immune‐restoring strategies in CAD.

## Introduction

The presence of an immune‐mediated inflammatory response in atherosclerosis, involving both innate and adaptive immunity, is well established, but the many components of the immune system and the roles they play in human disease remain unclear 1. Accumulating evidence indicates that an adequate balance between pro‐ and anti‐inflammatory actions inhibits the destabilization of atherosclerotic plaques, whereas an imbalance favours destabilization. Regulatory T cells (Tregs) expressing the forkhead family transcription factor FOXP3 have a crucial role in the maintenance of immune balance. Tregs, which constitute a minor proportion of the total CD4^+^ cell population, are able to suppress pathogenic T cells 2, but also the innate immune response 3, 4. Low quantities or impaired function of Tregs have been reported in studies of systemic autoimmune disorders 5, 6 and a potential role of Treg deficiency in atherosclerosis has been suggested 7. Approaches to increase Treg numbers have led to reduced formation of atherosclerotic lesions in mouse models 8, 9. In addition, in a population‐based prospective study, Wigren *et al*. 10 recently demonstrated an association between low levels of CD4^+^FOXP3^+^ cells and increased risk of myocardial infarction indicating that Tregs are also atheroprotective in humans. In line with this, a reduction in the number of Tregs has been reported in patients with non‐ST elevation acute coronary syndrome (NSTE‐ACS) or unstable angina, whereas levels in patients with stable coronary artery disease (CAD) were similar to those in healthy subjects 11, 12, 13. Tregs have thus emerged as an attractive therapeutic target to treat or prevent ACS. However, to better understand the role of Tregs in CAD, it is important to consider recent developments in defining subpopulations of Tregs as well as their functional properties.

The circulating pool of Tregs represents a heterogeneous population that is maintained through a homeostatic and self‐correcting process 2, 14, 15. In adults, around one‐third of the total Treg population consists of a naïve CD45RA^+^FOXP3^low^ phenotype (nTregs). The other two‐thirds consist? of a memory‐like CD45RA^−^FOXP3^high^ Treg phenotype (mTregs) that either originates from recently activated nTregs or regenerates in the periphery 14, 15, 16. Of note, FOXP3 is expressed in recently activated but non‐suppressive T cells, which has biased previous findings. The use of CD45RA/FOXP3 or CD45RA/CD127 gating ensures that ‘false‐positive Tregs’ (CD45RA^−^FOXP3^low^) are not defined as suppressive Tregs 15. Due to thymic involution, the proportion of nTregs normally declines with age, whilst the proportion of mTregs shows a reciprocal increase. However, different patterns in the balance between nTregs and mTregs have been observed in chronic inflammatory diseases 6, 15, 17. To date, only one previous study has investigated the composition of Tregs in patients with CAD, and the results suggested an imbalance of Treg subsets in those with ACS 13.

The phenotypical heterogeneity of Tregs may also imply differences in function. Studies of Treg function in autoimmune diseases have, however, yielded disparate results, and it has been suggested that this may be explained at least partly by variations in the composition of the Treg population 6, 15. Except for a compromised suppressive capacity in the total Treg compartment, shown in one previous study by Mor *et al*. 11, the Treg function is mainly unknown in CAD; the consequences of a Treg deficit in terms of inflammation and plaque occurrence also remain unclear.

The aim of the present study was to characterize the Treg composition in patients with CAD by performing detailed phenotypic and functional analyses of nTregs and mTregs separately. We found that Treg homeostasis was impaired in both NSTE‐ACS and post‐ACS patients compared with healthy subjects. Furthermore, lower replicative rates and compromised suppressive functions were observed in nTregs and mTregs from patients. The Treg defect was associated with a proinflammatory cytokine profile and increased occurrence of plaque in carotid arteries, thus highlighting its physiological relevance.

## Materials and methods

### Study population

Twenty‐six patients with ACS, 57 post‐ACS patients and 41 healthy control subjects were consecutively recruited at the Department of Cardiology, University Hospital, Linköping, Sweden. All patients in the ACS group had a diagnosis of NSTE‐ACS. Blood was collected within 24 h after admission [mean 15 (interquartile range 8–20) h] and always prior to coronary angiography. In the post‐ACS group, 24 (42%) had suffered from ST elevation (STE)‐ACS and 33 (58%) from NSTE‐ACS. Blood was collected between 6 and 12 months after the ACS event. For the control group, individuals residents of Linköping were randomly selected from the Swedish Population Register and invited to participate in the study. Individuals who accepted the invitation were included as controls if they were anamnestically healthy. Use of statins or antihypertensive drugs for primary disease prevention was allowed in the control group.

Patients were not eligible if they were more than 75 years of age, suffered from severe heart failure, immunological disorders, neoplastic disease, had evidence of acute or recent (<2 months) infection or major trauma, had undergone surgery or a revascularization procedure or received treatment with immunosuppressive or anti‐inflammatory agents (except low‐dose aspirin). The study was conducted in accordance with the ethical guidelines of the Declaration of Helsinki, and the research protocol was approved by the Ethical Review Board of Linköping. Written informed consent was obtained from all study participants.

### Carotid B‐mode ultrasound

In post‐ACS patients and control subjects, intima–media thickness (IMT) and the occurrence of plaques in both carotid arteries were assessed by B‐mode ultrasound using a 9–18 MHz linear two‐dimensional transducer (ACUSON S2000 TM ultrasound system; Siemens Medical Solutions USA, Inc., Mountain View, CA, USA).

Intima–media thickness was measured in the common carotid artery 1 cm below the bifurcation of the external and internal carotid arteries. A mean value of IMT was determined from two measurements. Plaques were defined according to the Mannheim Consensus 18 as structures 50% thicker than IMT in the focal area or a thickness >1.5 mm.

### Blood samples

Peripheral venous blood was collected from all subjects in the morning. Peripheral blood mononuclear cells (PBMCs) were isolated by gradient centrifugation using Lymphoprep (Axis‐Shield, Oslo, Norway). Plasma was obtained after centrifugation and stored at −80 °C.

### Flow cytometry analysis of total Tregs and Treg subsets in whole blood

The following monoclonal antibodies were used for analysis of total Tregs and Treg subpopulations in whole blood: anti‐CD3‐PerCP/Horizon (clone SK7/UCHT1), anti‐CD4‐PerCP/APC‐H7 (clone SK37/SK3), anti‐CD25‐Pe‐Cy7 (clone 2A3), anti‐CD45RA‐FITC (clone L48), anti‐CTLA‐4‐APC (clone BN13), anti‐CD39‐APC (clone TU66), anti‐HLA‐DR‐APC‐H7 (clone L243) and anti‐FOXP3‐PE (clone PCH101). All monoclonal antibodies were purchased from BD Biosciences (San José, CA, USA). For surface staining, cells were incubated with antibodies for 15 min at room temperature. After lysis of red blood cells, leucocytes were washed, fixed and permeabilized followed by intracellular staining of FOXP3 for 15 min at room temperature. Cells were analysed using a FACSCanto II flow cytometer (BD Biosciences). Control of the instrument settings was done each day using either seven‐colour Setup Beads with software (facsdiva software, BD Biosciences) or (Cytometer Setup and Tracking Beads) with (Cytometer Setup and tracking software, BD Biosciences). A minimum of 10 000 lymphocytes was acquired/recorded for data analyses. kaluza analysis Software (Beckman Coulter, Brea, CA, USA) was used for data analysis.

### Isolation and flow cytometry analysis of sorted nTregs and mTregs

CD4^+^ T cells were first isolated from PBMCs by negative selection using CD4^+^ T Cell Isolation Kit II (Miltenyi Biotec, Bergisch Gladbach, Germany). Unlabelled CD4^+^ T cells were then stained with Human Regulatory T cell Sorting Cocktail (CD45RA‐FITC, CD127‐Alexa Fluor647, CD25‐PE and CD4‐PerCP‐Cy5.5) (BD Biosciences). Next, stained cells were washed and separated into CD45RA^+^CD4^+^CD25^high^CD127^−/low^ (nTregs), CD45RA^−^CD4^+^CD25^high^CD127^−/low^ (mTregs) and CD4^+^CD25^−^CD127^+^ T cells (responder T cells) using a FACSAria cell sorter (BD Biosciences). The cell subsets were sorted at a rate of 10 000 events s^−1^, washed and resuspended into different fractions. One fraction was stained with anti‐FOXP3‐Horizon V450 and/or IgG1 isotype‐Horizon V450 (BD Biosciences) and analysed by flow cytometry, as described above. To evaluate FOXP3 expression per cell, mean fluorescence intensity (MFI) was determined by dividing the geometric MFI for FOXP3^+^ cells by the geometric mean MFI for the corresponding FOXP3 isotype controls.

### FOXP3 mRNA expression in sorted nTregs and mTregs

Total RNA was extracted from fractions of nTregs and mTregs using innuPrep RNA kit (Analytik Jena, Thuringia, Germany). The quantity and quality of total RNA was assessed using the Agilent 2100 Bioanalyzer (Santa Clara, CA, USA). cDNA was synthesized using the High Capacity cDNA Reverse Transcription kit with RNase Inhibitor (Invitrogen, Paisley, UK). PCR preamplification of cDNA for FOXP3 and the endogenous control GAPDH was performed using TaqMan PreAmp Master Mix (Applied Biosystems, Warrington, UK). Briefly, *FOXP3* and *GAPDH* TaqMan gene expression assays were pooled and 12.5 μL of the pooled assay mix was added to 12.5 μL of each cDNA sample and 25 μL of the TaqMan PreAmp Master Mix in a final volume of 50 μL. The preamplification PCR conditions were as follows: initial hold at 95 °C for 10 min and 10 preamplification cycles of 15 s at 95 °C and 4 min at 60 °C. Next, the preamplified products were diluted 1 : 4. Quantitative real‐time PCR amplifications were performed with FAM‐labelled TaqMan primer/probe sets for FOXP3 (Hs01085834_m1) and GAPDH (Hs03929097_g1) as internal control (Life Technologies, Paisley, UK) using 1 μL of the preamplified cDNA. Samples were run in a 384‐well format plate using the ABI PRISM 7900HT fast real‐time system (Applied Biosystems). FOXP3 mRNA expression levels in nTregs and mTregs were normalized and quantified relative to GAPDH expression according to Sequence Detector User Bulletin 2 (Applied Biosystems).

### Functional assays of nTregs and mTregs

Ninety‐six‐well plates were coated with anti‐CD3/anti‐CD28 antibodies (AbD Serotec, Oxford, UK) as previously described 19. Treg subsets (nTregs or mTregs) were cultured with responder T cells (CD4^+^CD25^−^CD127^+^) for 3 days separately or together (ratio of 1 : 1). Proliferation was assessed using a colourimetric immunoassay based on bromo‐2′‐deoxyuridine incorporation during DNA synthesis (Roche, Mannheim, Germany). Suppression was calculated using the following formula: % suppression = [(T responder cell proliferation without Tregs − T responder cell proliferation with Tregs)/(T responder cell proliferation without Tregs)] × 100.

In a parallel series of experiments, supernatants were harvested for analysis of cytokines after 72 h of culture. Interferon (IFN)‐γ and IL‐10 levels were measured using a Fluorokine Map Human kit (R&D system, Abingdon, UK), and thereafter, plates were read with a luminex SD instrument (Invitrogen). The lowest detection limits were 0.08 pg mL^−1^ for IFN‐γ and 0.24 pg mL^−1^ for IL‐10.

### Cytokine production by PBMCs

Peripheral blood mononuclear cells from post‐ACS patients and control subjects were washed twice in phosphate‐buffered saline and resuspended at a concentration of 1 × 10^6^ mL^−1^ in RPMI‐1640 medium (Gibco Invitrogen, Carlsbad, CA, USA) supplemented with L‐glutamine (Gibco), 10% FCS, 100 U mL^−1^ penicillin and 100 μg mL^−1^ streptomycin (Gibco). The cells were left to incubate for 19 h at 37 °C with or without 100 ng mL^−1^ endotoxin‐free lipopolysaccharide (LPS) from *Escherichia coli* (Sigma‐Aldrich, St Louis, MO, USA). Cell supernatants were then removed and IL‐1β, IL‐6 and IL‐10 concentrations were measured using Luminex human premixed multi‐analyte kits (R&D systems, Abingdon, UK). The standard curve ranges were 2.7–1987 for IL‐1β, 3.0–2223 for IL‐6 and 6.1–4474 pg mL^−1^ for IL‐10; the interassay coefficients of variation (CV) were 12.5%, 18.3% and 9.9%, respectively.

### Cytokines in plasma

IL‐6 and IL‐10 were analysed in EDTA‐anticoagulated plasma using Human IL‐6 QuantiGlo enzyme‐linked immunosorbent assay (ELISA) chemiluminescence kit and IL‐10 Quantikine HS ELISA kit (R&D Systems). The lowest limits of detection were 0.48 pg mL^−1^ for IL‐6 and 0.17 pg mL^−1^ for IL‐10; the interassy CVs were 11.6% and 25%, respectively.

### Statistical analyses

ibm spss statistics 20 (SPSS Inc., Chicago, IL, USA) was used for all statistical analyses.

The chi‐squared and Mann–Whitney *U*‐tests were used for analyses between two groups. Kruskal–Wallis analysis with *post hoc* Dunn's test was used for comparison between three groups. Differences between related samples were analysed by Friedman's or Wilcoxon signed‐rank tests. Bivariate correlations were analysed by Spearman's rank correlation coefficient. *P*‐values <0.05 were considered statistically significant.

## Results

### Characteristics of patients and controls

The characteristics of NSTE‐ACS and post‐ACS patients and control subjects are shown in Table 1. There were no differences in age, sex or body mass index between groups; however, some parameters such as statin use and lipid profile differed significantly. The NSTE‐ACS group had higher levels of IL‐6 in plasma compared with the post‐ACS and control groups. IL‐10 levels in plasma did not differ across groups but were in general very low (and nondetectable in 29% of the samples).

**Table 1:** Characteristics of control subjects and post‐ACS and NSTE‐ACS patients

	Controls *n* = 41	Post‐ACS *n* = 57	NSTE‐ACS *n* = 26	*P*‐value
Age, years	67 (66–72)	66 (61–71)	68 (61–73)	NS
Female, *n* (%)	11 (27)	12 (21)	8 (31)	NS
BMI, kg m^−2^	26 (25–28)	27 (25–30)	26 (24–31)	NS
Smoking, *n* (%)	1 (2)	6 (11)	3 (12)	NS
Diabetes, *n* (%)	0 (0)	12 (21)	8 (31)	NS
Hypertension, *n* (%)	5 (12)	28 (49)	16 (62)	NS
Statin use, long term/short term^a^, *n* (%)	4 (9.7)/0(0)	54 (95)/0 (0)	8 (31)/9 (35)	<0.001
Arterial imaging				
Coronary angiography 0‐/1‐/2‐/3‐vessel disease^b^, *n*	–	2/21/16/18	0/10/5/11	NS
IMT, right CCA, mm	0.78 (0.70–0.95)	0.80 (0.70–0.95)	–	NS
IMT, left CCA, mm	0.80 (0.70–0.90)	0.75 (0.65–0.95)	–	NS
Presence of plaques in right carotid artery, *n* (%)	21/41 (51)	44 (77)	–	<0.01
Presence of plaques in left carotid artery, *n* (%)	24/41 (59)	36 (63)	–	NS
Plasma measurements				
Total cholesterol, mmol L^−1^	5.4 (4.5–5.9)	3.9 (3.2–4.3)	5.0 (4.2–6.2)	<0.001
LDL cholesterol, mmol L^−1^	3.1 (2.5–4.0)	2.0 (1.7–2.4)	2.6 (2.1–3.4)	<0.001
HDL cholesterol, mmol L^−1^	1.6 (1.4–1.8)	1.1 (0.9–1.4)	1.3 (1.0–2.0)	<0.001
Triglycerides, mmol L^−1^	1.1 (0.8–1.5)	1.2 (0.9–1.7)	1.4 (0.8–2.9)	NS
IL‐6, pg mL^−1^	2.5 (1.3–3.1)	2.4 (1.8–3.3)	4.8 (2.6–8.8)	<0.01
IL‐10, pg mL^−1^	0.31 (0.26–0.43)	0.31 (0.24–0.40)	0.36 (0.25–0.55)	NS
Troponin T, ng L^−1^ c	–	–	79 (39–132)	

BMI, body mass index; CCA, common carotid artery; IMT, intima–media thickness; ACS, acute coronary syndrome; NSTE‐ACS, non‐ST elevation acute coronary syndrome.

Data are presented as median (interquartile range), unless otherwise stated.

^a^Statin use: long term, simvastatin or atorvastatin >1 month; short term, high‐dose atorvastatin administered 1 day prior to coronary angiography. ^b^Based on number of significantly stenosed vessels (>70%). ^c^Maximum troponin T test result in the first 12 h.

### Impaired Treg homeostasis in NSTE‐ACS and post‐ACS patients

**Figure 1:**
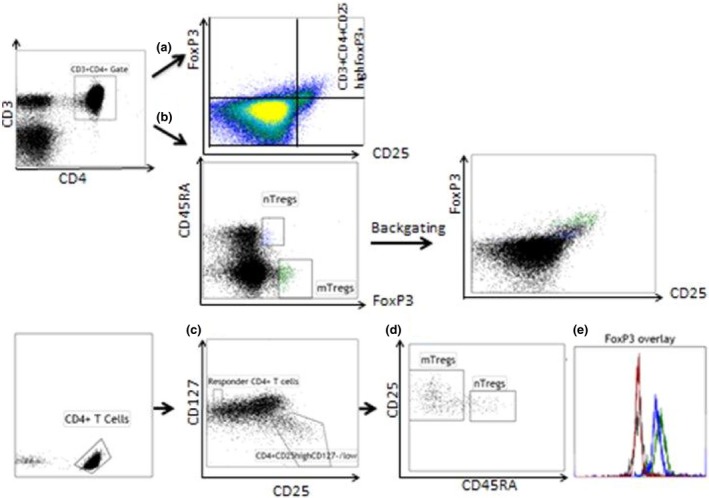
Gating strategies for total regulatory T cells (Tregs) and Treg subsets in blood and isolated CD4^+^ T cells. Lymphocytes were gated by forward and side scatter, followed by expression of CD3 and CD4. Cells were further analysed by expression of CD25^high^ and FOXP3 for identification of total Tregs as CD3^+^ CD4^+^ CD25^high^ FOXP3^+^ (a) or expression of FOXP3 and CD45RA resulting in CD45RA ^+^ [naïve (n)] and CD45RA ^−^ [memory (m)] CD3^+^ CD4^+^ FOXP3^+^ Treg subsets (b). Isolated CD4^+^ T cells were gated by expression of CD4 followed by expression of CD25 and CD127 (c). CD4^+^ CD25^−^ CD127^+^ cells were defined as T responder cells. The total Treg population (CD4^+^ CD25^high^ CD127^−/low^) was divided into nTregs (CD45RA ^+^) and mTregs (CD45RA ^−^) (d). Each fraction was stained for FOXP3 and isotype control, as shown in the histogram (e): FOXP3 isotype control (black), FOXP3 expression in T responder cells (red), FOXP3 expression in nTregs (blue) and mTregs (green).

As shown in Table 2, the absolute numbers of CD3^+^CD4^+^ T cells did not differ between groups, whereas the proportions of CD3^+^CD4^+^ T cells expressing the late activation marker HLA‐DR were higher in both NSTE‐ACS and post‐ACS patients compared with control subjects. The proportions of all naïve T cells (CD45RA^+^) within the CD4^+^ population were significantly lower in post‐ACS patients and tended to be lower in NSTE‐ACS patients, compared with control subjects. The proportions of total Tregs in whole blood and isolated CD4^+^ T cells were determined by different gating strategies, as shown in Fig. 1. In whole blood, the total Treg population was identified as CD3^+^CD4^+^CD25^high^FOXP3^+^ cells, and in isolated CD4^+^ T cells using the combination of CD4, CD25 and CD127. Both strategies showed that the proportions of total Tregs were significantly decreased in NSTE‐ACS and post‐ACS patients compared with control subjects (Table 2). The subsets of nTregs and mTregs were identified using CD45RA in combination with FOXP3 (whole blood) or CD45RA in combination with CD25^high^ and CD127^−/low^ (isolated CD4^+^ T cells), which allowed the subdivision into CD45RA^+^ Tregs (nTregs) and CD45RA^−^ Tregs (mTregs). Independent of chosen strategy, the proportions of nTregs were consistently lower in NSTE‐ACS and post‐ACS patients than in control subjects. In addition, the proportions of mTregs in blood were significantly lower in patients compared with control subjects, whilst proportions of mTregs in isolated CD4^+^ T cells did not differ between groups. To take into account the interindividual variation in each group, we also performed one‐way anova. This analysis showed similar results to those of the nonparametric tests (data not shown).

**Table 2:** Proportions of total Tregs, nTregs and mTregs based on different gating strategies in whole blood and isolated CD4^+^ T cells of control subjects and post‐ACS and NSTE‐ACS patients

	Controls *n *=* *41	Post‐ACS *n *=* *57	NSTE‐ACS *n *=* *26	*P*‐value
CD3^+^CD4^+^ T cells, cells μL^−1^	938 (779–1168)	837 (605–1055)	913 (586–1368)	NS
CD3^+^CD4^+^HLA‐DR^+^, % of CD4^+^	2.2 (1.4–3.1)	2.9 (2.1–3.5)^c^	3.3 (2.2–5.9)^b^	0.01
CD3^+^CD4^+^CD45RA^+^, % of CD4^+^	32 (23–41)	25 (18–39)^b^	26 (17–38)^b^	NS (0.066)
Total Tregs				
CD3^+^CD4^+^CD25^high^FOXP3^+^, % of CD4^+^ in blood	4.2 (3.2–5.3)	3.7 (2.9–4.7)^c^	3.3 (2.9–4.0)^b^	<0.05
CD3^+^CD4^+^CD25^high^CD127^−/low^, % of isolated CD4^+^	6.5 (6.0–7.6)	5.8 (5.2–6.7)^b^	5.0 (4.3–5.9)^a^	<0.01
nTregs				
CD3^+^CD4^+^ FOXP3^+^CD45RA^+^,% of CD4^+^ in blood	1.1 (0.9–1.5)	0.6 (0.5–0.8)^a^	0.4 (0.3–0.7)^a^^,^ d	<0.001
CD3^+^CD4^+^CD25^high^CD127^−/low^ CD45RA^+^, % of isolated CD4^+^	1.9 (1.7–2.4)	1.1 (0.7–1.4)^a^	1.2 (0.5–1.7)^a^	<0.001
mTregs				
CD3^+^CD4^+^ FOXP3^+^CD45RA^−^,% of CD4^+^ in blood	3.1 (2.5–3.7)	2.6 (2.3–3.0)^b^	2.4 (2.0–2.9)^a^	<0.001
CD3^+^CD4^+^CD25^high^CD127^−/low^ CD45RA^−^, % of isolated CD4^+^	4.3 (3.8–5.3)	4.5 (3.5–5.1)	4.1 (3.4–4.4)	NS

Treg, regulatory T cell; n, naïve; m, memory; ACS, acute coronary syndrome; NSTE‐ACS, non‐ST elevation acute coronary syndrome.

Data are presented as median (interquartile range).

^a^ *P* < 0.001 versus controls. ^b^ *P* < 0.01 versus controls. ^c^ *P* < 0.05 versus controls. ^d^ *P* = 0.070 versus controls.

There were no significant differences in Treg composition between patients who received low‐to‐moderate‐intensity (*n *=* *39) or high‐intensity statin therapy (*n *=* *23). Neither were there any differences in Tregs between users [long‐term (*n *=* *8) or short‐term use (*n *=* *9)] and nonusers of statins (*n *=* *9) within the NSTE‐ACS group. Furthermore, the Treg composition within the post‐ACS group was unaffected by whether the index event 6–12 months earlier was NSTE‐ACS or STE‐ACS.

### Expression of CTLA‐4 and CD39 within Treg subsets

**Figure 2:**
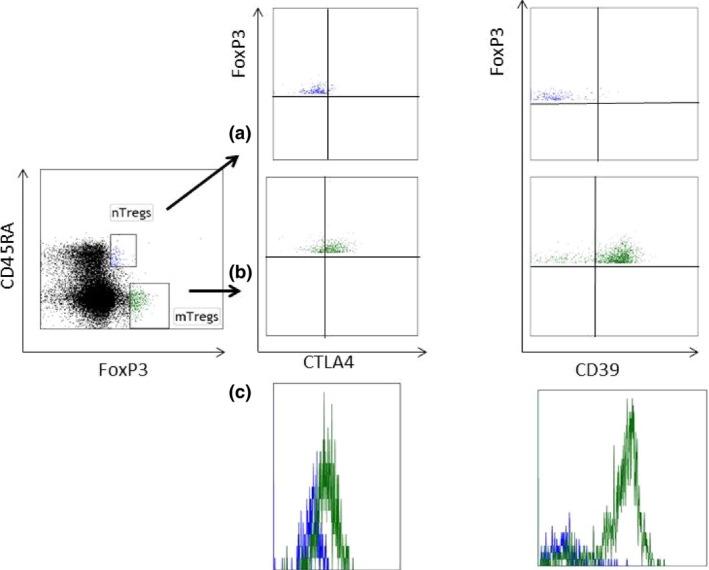
Expression of CTLA4 and CD39 in naïve (n)Tregs and memory (m)Tregs in blood. A representative flow cytometry plot is shown for the staining of CTLA4 and CD39 in nTregs (a) and mTregs (b). The overlapping histograms of CTLA4 and CD39 in nTregs (blue) and mTregs (green) are also shown (c).

The expression of CTLA‐4 and CD39 has been associated with Treg differentiation and function 2. As expected, CTLA‐4 and CD39 were abundantly expressed in mTregs, whereas expression levels of both were much lower in nTregs (Fig. 2). In both NSTE‐ACS and post‐ACS patients, the proportions of CTLA‐4^+^ and CD39^+^ nTregs were significantly increased compared with control subjects (Table 3).

**Table 3:** Expression of CTLA‐4 and CD39 in nTregs and mTregs from control subjects and post‐ACS and NSTE‐ACS patients

	Controls *n *=* *41	Post‐ACS *n *=* *57	NSTE‐ACS *n *=* *26	*P*‐value
nTregs, CD3^+^CD4^+^ FOXP3^+^CD45RA^+^				
CTLA‐4^+^, %	7.2 (4.3–8.7)	8.7 (7.4–10)^b^	9.6 (7.9–12)^a^^,^ d	<0.001
CD39^+^, %	1.5 (1.0–2.1)	2.1 (1.1–3.3)^b^	3.4 (2.0–4.9)^a^^,^ c	<0.001
mTregs, CD3^+^CD4^+^ FOXP3^+^CD45RA^−^				
CTLA‐4^+^, %	87 (83–89)	85 (83–89)	85 (84–87)	NS
CD39^+^, %	77 (57–82)	78 (64–83)	74 (44–83)	NS

Treg, regulatory T cell; n, naïve; m, memory; ACS, acute coronary syndrome; NSTE‐ACS, non‐ST elevation acute coronary syndrome.

Values indicate % of positively stained cells in nTreg and mTreg fractions in whole blood. Data are presented as median (interquartile range).

^a^ *P* < 0.001 versus controls. ^b^ *P* < 0.01 versus controls. ^c^ *P* < 0.01 vs post‐ACS. ^d^ *P* = 0.077 vs post‐ACS.

### Lower proportions of FOXP3^+^ Treg subsets but higher expression of FOXP3 at the single‐cell level in NSTE‐ACS and post‐ACS patients

The analysis of FOXP3 expression in sorted nTreg and mTreg populations revealed significant differences between patients and control subjects (Table 4). In general, the proportions of FOXP3^+^ cells amongst nTregs and mTregs were high, although significantly lower in both NSTE‐ACS and post‐ACS patients compared with controls. The FOXP3 protein expression per cell, expressed as geometric mean MFI, was significantly lower in nTregs than in mTregs (*P *<* *0.001), which is in line with previous findings showing that nTegs and mTregs are FOXP3^low^ and FOXP3^high^, respectively 15. However, in both nTregs and mTregs, the FOXP3 MFI was significantly higher in patients than in control subjects, with the highest expression in patients with NSTE‐ACS. The FOXP3 mRNA levels were also higher in nTregs from patients, whereas mRNA levels in mTregs did not differ between groups. Within the responder T‐cell population, the proportions of FOXP3^+^ cells were equally low in all groups, whereas the FOXP3 MFI levels were significantly higher in patients, particularly the ACS group.

**Table 4:** FOXP3 expression in nTregs and mTregs in control subjects and post‐ACS and NSTE‐ACS patients

	Controls *n *=* *41	Post‐ACS *n *=* *57	NSTE‐ACS *n *=* *26	*P*‐value
nTregs, CD3^+^CD4^+^CD25^high^CD127^−/low^CD45RA^+^				
FOXP3^+^, % of nTregs	90 (83–97)	83 (67–93)^c^	83 (56–90)^c^	<0.05
FOXP3, MFI	21 (20–26)	30 (23–35)^a^	41 (31–52)^a^^,^ e	<0.001
FOXP3, mRNA	1.38 (0.63–2.23)	3.41 (2.67–4.05)^b^	2.84 (1.69–4.02)^c^	<0.01
mTregs, CD3^+^CD4^+^CD25^high^CD127^−/low^CD45RA^−^				
FOXP3^+^, % of mTregs	94 (88–96)	85 (68–92)^b^	82 (68–92)^b^	<0.01
FOXP3, MFI	32 (28–38)	37 (30–43)^c^	46 (41–51)^a^^,^ e	<0.001
FOXP3, mRNA	0.88 (0.77–1.41)	1.16 (1.01–1.37)	0.96 (0.59–1.17)	NS
Responder T cells, CD3^+^CD4^+^CD25^−^CD127^+^				
FOXP3, % of responder T cells	1.7 (0.9–4.5)	1.9 (0.8–13)	2.4 (0.9–13)	NS
FOXP3, MFI	18 (14–27)	26 (20–31)^c^	36 (31–50)^a^^,^ d	<0.001

Treg, regulatory T cell; n, naïve; m, memory; ACS, acute coronary syndrome; NSTE‐ACS, non‐ST elevation acute coronary syndrome.

Data are presented as median (interquartile range).

^a^ *P* < 0.001 versus controls. ^b^ *P* < 0.01 versus controls. ^c^ *P* < 0.05 versus controls. ^d^ *P* < 0.001 versus post‐ACS. ^e^ *P* < 0.01 versus post‐ACS.

### Lower proliferative capacity and impaired function of nTregs and mTregs in NSTE‐ACS and post‐ACS patients

**Figure 3:**
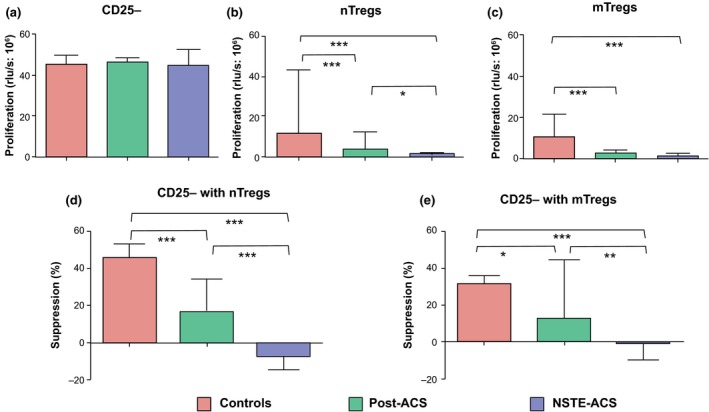
Proliferation assays were performed in samples from control subjects (*n *=* *23) and post‐acute coronary syndrome (ACS;* n *=* *20) and non‐ST elevation (NSTE)‐ACS (*n *=* *17) patients. Cells were stimulated with plate‐bound anti‐CD3/CD28 for 3 days. Proliferation was assessed using a bromo‐2′‐deoxyuridine assay and expressed as relative light units (rlu) s^−1^. Proliferation rates CD25^−^ T responder cells (a), naïve (n) regulatory T cells (Tregs) (b) and memory (m)Tregs (c). Percentage suppression of proliferation when CD25^−^ T responder cells were cocultured (ratio of 1 : 1) with nTregs (d) or mTregs (e). Data are presented as median (interquartile range). Between‐group differences: *P <* *0.05, **P <* *0.01, ***P <* *0.001.

The proliferative and functional properties of isolated Treg fractions were evaluated in subgroups of patients and control subjects by inducing T‐cell receptor (TCR)‐mediated activation in a system with plate‐bound anti‐CD28 and anti‐CD3. As shown in Fig. 3, the proliferative response of responder T cells was similar across groups. Compared with responder T cells, isolated nTregs and mTregs exhibited markedly lower proliferative rates, however, with significant differences between patients and controls. The proliferation of nTregs was lower in both patient groups compared with the control group, and also lower in NSTE‐ACS compared with post‐ACS patients. The proliferation of mTregs was lower in all patients, irrespective of group (NSTE‐ACS or post‐ACS). The proliferation of nTregs correlated positively with the proportions of nTregs (*r *=* *0.312, *P *<* *0.01), and negatively with FOXP3 MFI of nTregs (*r *=* *−0.382, *P *<* *0.001). Furthermore, the proliferation of mTregs correlated negatively with FOXP3 MFI of mTregs (*r *=* *−0.311, *P *<* *0.01). When nTregs or mTregs were cocultured with responder T cells (ratio of 1 : 1), the capacity to suppress proliferation was significantly impaired in patients, particularly in the NSTE‐ACS group (Fig. 3). This method does not allow the discrimination between responder T‐cell and Treg proliferation. However, the capacity to suppress proliferation correlated with the proliferation of each Treg subset alone (nTreg, *r* = 0.408; mTreg, 0.386; both *P* < 0.001).

**Figure 4:**
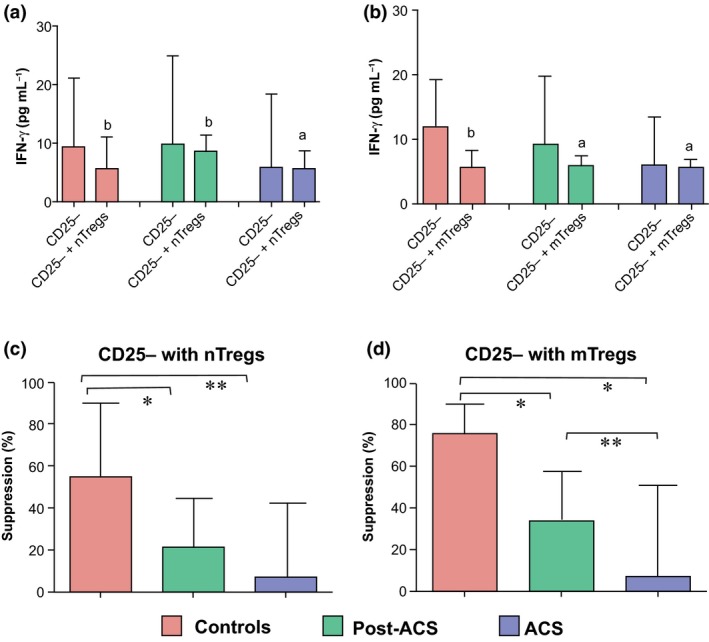
Interferon‐gamma (IFN‐γ) secretion assays were performed in samples from control subjects (*n *=* *10) and post‐acute coronary syndrome (ACS;* n *=* *10) and non‐ST elevation (NSTE)‐ACS (*n *=* *9) patients. Cells were stimulated with plate‐bound anti‐CD3/CD28 for 3 days and IFN‐γ was measured in the supernatant. Secretion of IFN‐γ in T responder cells alone and in cocultures of T responder cells (ratio of 1 : 1) with naïve (n) regulatory T cells (Tregs) (a) or memory (m)Tregs (b). Percentage suppression of IFN‐γ secretion in cocultures with nTregs (c) and mTregs (d). Data are presented as median (interquartile range). Within‐group differences (a and b): ^a^ *P* < 0.05, ^b^ *P* < 0.01; between‐group differences (c and d): *P <* *0.05, **P <* *0.01.

**Figure 5:**
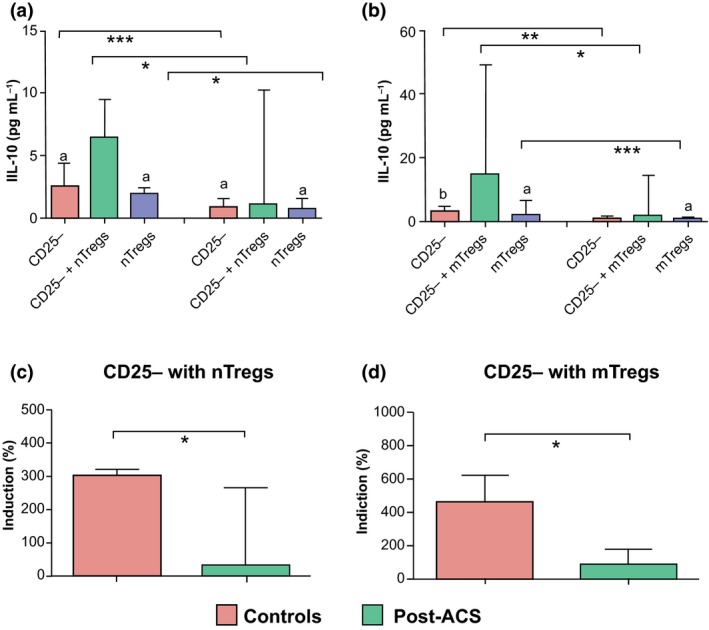
IL‐10 secretion assays were performed in samples from control subjects (*n *=* *10) and post‐acute coronary syndrome (ACS) patients (*n *=* *9). Cells were stimulated with plate‐bound anti‐CD3/CD28 for 3 days and IL‐10 was measured in the supernatant. Secretion of IL‐10 in T responder cells alone, coculture of T responder cells with nTregs (1 : 1) and nTregs alone (a), in T responder cells alone, cocultures of T responder cells with mTregs (1 : 1) and mTregs alone (b). Percentage increase in IL‐10 secretion in cocultures of T responder cells with nTregs (c) and mTregs (d). Data are presented as median (interquartile range). Within‐group differences (a and b): ^a^ *P* < 0.05, ^b^ *P* < 0.01. Between‐group differences (a–d): *P <* *0.05, **P <* *0.01, ***P <* *0.001.

This system with plate‐bound anti‐CD28 and anti‐CD3 was also used to study the secretion of IFN‐γ and IL‐10. The secretion of IFN‐γ by responder T cells was similar in patients and control subjects. In supernatants from Treg subsets, IFN‐γ was below the detection limit of 0.08 pg mL^−1^. When nTregs or mTregs were cocultured with responder T cells (ratio of 1 : 1), the capacity to inhibit secretion of IFN‐γ was significantly lower in NSTE‐ACS and post‐ACS patients compared with control subjects (Fig. 4). As shown in Fig. 5, the secretion of IL‐10 by responder T cells, nTregs or mTregs alone was significantly higher in control subjects than in post‐ACS patients; levels were always below the detection limit of 0.24 pg mL^−1^ in NSTE‐ACS patients. When nTregs or mTregs were cocultured with responder T cells (ratio of 1 : 1), the capacity to secrete IL‐10 was significantly enhanced, particularly in controls. The capacity of Tregs to secrete IL‐10 in monocultures correlated with the proliferative rate of each Treg subset alone (nTreg, *r* = 0.408; mTreg, 0.386; both *P* < 0.001).

### Impaired function of nTregs and mTregs is associated with LPS‐induced proinflammatory responses

**Figure 6:**
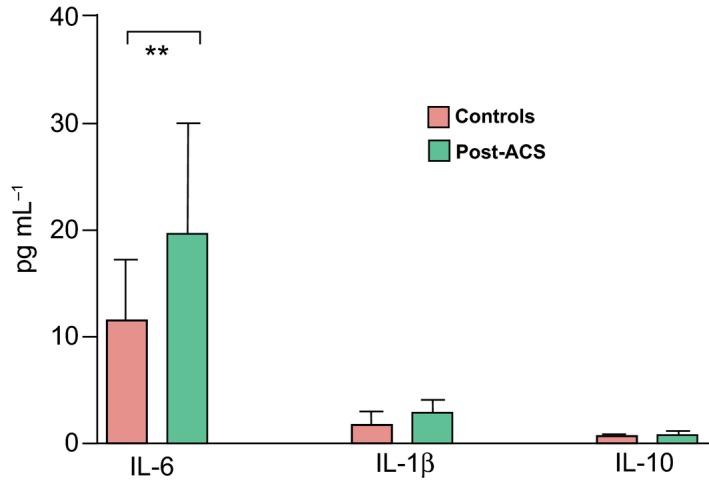
Peripheral blood mononuclear cells from control subjects (*n *=* *31; white columns) and post‐acute coronary syndrome (ACS) patients (*n *=* *54; grey columns) were incubated with endotoxin‐free lipopolysaccharide for 19 h at 37 °C. Secretion of IL‐6, IL‐1β and IL‐10 into the supernatant is shown. Data are expressed as median (interquartile range). Between‐group difference: **P <* *0.01. There was a trend towards a significant difference for IL‐1β levels: *P *=* *0.084.

To investigate the potential consequences of the impaired Treg function, we assessed the correlation between Treg functional capacity and LPS‐induced cytokine response in post‐ACS patients and control subjects. The secretion of IL‐6 was significantly higher and IL‐1β tended to be higher (*P *=* *0.084) in post‐ACS patients, whereas the secretion of IL‐10 did not differ between groups (Fig. 6). The secretion of IL‐6 and IL‐1β from LPS‐stimulated PBMCs was inversely correlated with the capacity of nTregs to suppress proliferation (*r *=* *−0.396 and −0.277, *P *<* *0.01 and <0.05, respectively), and also with the secretion of IL‐10 from TCR‐stimulated nTregs (*r *=* *−0.602 and −0.617, respectively) and mTregs (*r *=* *−0.529 and −0.578, respectively; all *P *<* *0.01).

### A low proportion of nTregs is associated with the presence of plaques in carotid arteries

The carotid IMT on both sides and the presence of plaques in the left carotid were similar in post‐ACS patients and control subjects, whereas plaques in the right carotid were more common in patients (Table 1). The majority of carotid plaques caused nonsignificant (<50%) stenosis; a stenosis greater than 50% of the luminal diameter was only observed in six patients. The proportions of nTregs were inversely correlated with the presence of plaques on the right (*r *=* *−0.288, *P *<* *0.01) and left sides (*r *=* *−0.219, *P *<* *0.05). In addition, the nTreg levels were inversely correlated with right carotid IMT (*r *=* *−0.249, *P *<* *0.05). There were no correlations between the proportions of mTregs or total Tregs and IMT or plaque occurrence.

## Discussion

In the present study, we identified total Tregs and Treg subsets in peripheral blood and isolated CD4^+^ T cells using an updated set of validated Treg markers. The proportions of total Tregs and nTregs, both in blood and in isolated CD4^+^ T cells, were significantly lower in NSTE‐ACS patients compared with control subjects, which is in line with previous studies showing low Treg levels in the early phase of NSTE‐ACS 11, 12, 13. A significant reduction in Tregs, although less pronounced, was also noted in stable post‐ACS patients, which was less expected as it has been reported that Treg levels in patients with stable CAD are comparable to those in control subjects 11, 12, 13. This discrepancy may be attributed to differences in flow cytometry‐based methods of identification of Tregs, but also to heterogeneity in patient populations as former studies have mainly included stable CAD patients without a history of ACS. There has been speculation regarding whether the reduction in Tregs in NSTE‐ACS is mainly a cause or a consequence of the acute event; so far, evidence suggests that it can be both. As recently demonstrated by Wigren *et al*. 10, individuals with low levels of Tregs are at increased risk of a first coronary event. Our findings indicate that reduced levels of Tregs persist up to at least 12 months after an acute event. In agreement with this, George *et al*. 20 reported that patients with a history of recurrent ACS had lower Treg levels than CAD patients without a history of ACS despite similar coronary angiographic findings. On the other hand, Ammirati *et al*. 21 performed longitudinal assessments in patients with ACS and found that Treg levels increased significantly after a median time of 55 days following the acute event, thus demonstrating the dynamics of Treg responses.

To elucidate the homeostatic mechanisms of Tregs, we also mapped Treg subpopulations in detail. In patients with CAD, our finding of a reduced nTreg fraction in combination with a decreased pool of circulating naïve CD4^+^ T cells may reflect a process of accelerated thymus involution. It is known that thymic involution is not only associated with ageing, causing nTreg levels to decline with age, but can also occur transiently during inflammation, infection and oxidative stress 22, 23. A state of downregulated thymic function in NSTE‐ACS patients was recently confirmed by Zhang *et al*. 13 who reported lower levels of CD45RA^+^ Tregs, including recent thymic emigrants expressing CD31, in patients with ACS but not in CAD patients without prior ACS. We found an increased expression of CTLA‐4 and CD39 in nTregs from both NSTE‐ACS and post‐ACS patients suggesting that these cells were undergoing accelerated conversion to mTregs, either as a compensatory mechanism for decreased thymic output or as a response to increasing demand in the periphery.

A homeostatic defect in CAD was evident as the low levels of nTregs in patients were not associated with any compensatory increase in mTregs. Instead, the levels of mTregs in peripheral blood were reduced as well, particularly in the NSTE‐ACS group, whereas no between‐group differences in mTreg levels were observed in isolated CD4^+^ T cells. In a previous study, the levels of CCR5^+^ Tregs were comparable in peripheral blood of NSTE‐ACS patients, stable patients with CAD and control subjects 21, thus supporting a possible lack of compensatory increase in mTregs. Although CCR5 does not conform to the current nomenclature of memory cells, it has been proposed that its expression is critical for mTreg function 24. A continuous proliferation of Tregs in the periphery is believed to be important for the maintenance of total Treg levels throughout life 2, 14, 15. We therefore investigated the proliferative potential *ex vivo* of Treg subsets and found reduced proliferation rates of nTregs and mTregs in both patient groups. Of interest, Carbone *et al*. 25 recently showed that Tregs from patients with multiple sclerosis had a lower proliferation rate both *in vivo* and *in vitro* compared with Tregs from healthy subjects and, moreover, that proliferation of Tregs was lower in patients with higher disease activity. These authors concluded that the impaired capacity of Tregs to proliferate might represent a mechanism underlying the reduced suppressive function and/or number of Tregs in autoimmune disorders. Accordingly, we found that the proliferation of nTregs *ex vivo* correlated with the proportions of nTregs *in vivo* and, not unexpectedly, the proliferation of Treg subsets correlated with their functional capacities *ex vivo*, including the secretion of IL‐10. In post‐ACS patients, the IL‐10‐secreting capacity was impaired also in responder T cells. In NSTE‐ACS patients, IL‐10 secretion from Tregs and responder T cells was nondetectable indicating a pronounced downregulation of IL‐10 during the acute event. This finding may be in line with the results of previous studies showing an enhanced proinflammatory CD4^+^ T‐cell response due to TCR‐signalling abnormalities in NSTE‐ACS patients 26, 27. It is interesting that this defect in TCR signalling was associated with impaired Treg differentiation 27. Data from several studies have also highlighted the imbalance between pro‐ and anti‐inflammatory cytokines in plaque instability and the importance of IL‐10 as a cytokine with atheroprotective effects 28, 29, 30.

Increased apoptosis related to inflammation and oxidative stress may contribute to the loss of Tregs. In particular, mTregs are susceptible to apoptosis 15. We did not assess apoptosis and can therefore only speculate that enhanced apoptosis of Tregs may have occurred in our patients. As recently shown by Zhang *et al*. 13, the total Treg population was more susceptible to apoptosis in NSTE‐ACS patients than in CAD patients without prior ACS. Another possible reason why mTreg levels in patients are not maintained or increased in blood is that they may have entered secondary lymphoid organs or sites of inflammation. However, investigating this hypothesis remains a challenge in atherosclerotic diseases. In a previous study investigating the distribution of Tregs in human atherosclerotic lesions, it was found that low numbers of Tregs were present during all developmental stages 31. We recently measured the proportions of Tregs in thoracic lymph nodes from patients undergoing coronary bypass surgery. The levels of total Tregs in lymph nodes were found to be twofold higher than in blood independent of whether patients had a diagnosis of ACS or stable CAD 32, but nTreg and mTreg fractions were not investigated. It remains a possibility that the low levels of circulating mTregs are at least partly due to a redistribution of these cells to lymphoid tissues.

Despite the low circulating levels and functional impairment of FOXP3^+^ Tregs in patients, we found that the FOXP3 protein expression was increased at the single‐cell level, particularly in NSTE‐ACS patients. In nTregs, this was also accompanied by a significant increase in FOXP3 mRNA expression. Because the conversion of nTregs to mTregs involves a conversion from FOXP3^low^ to FOXP3^high^ cells 2, the increased FOXP3 expression in nTregs may reflect an accelerated shift towards mTregs, as discussed above. Nonetheless, the finding of increased FOXP3 protein levels in nTregs and mTregs from patients is surprising because it has been consistently shown that FOXP3 expression in Tregs is correlated with suppressive functions, and suppressive capacities were consistently lower in patients in the present study. However, there is emerging evidence that FOXP3 expression in Tregs is not necessarily associated with a suppressive phenotype. By performing a single‐cell analysis of Tregs in healthy human donors, d'Hennezel *et al*. 33 showed that around 30% of CD25^high^FOXP3^+^ Tregs, preferentially residing in the CD45^−^ pool, were nonsuppressive. We also showed higher FOXP3 expression levels in T responder cells from patients, particularly the NSTE‐ACS group. This is compatible with an enhanced T‐cell activation *in vivo*, as it is known that CD4^+^ T effector cells transiently express FOXP3 upon activation but without gaining suppressive function 34. Accordingly, we found evidence for enhanced systemic activation of CD4^+^ T cells, assessed by the expression of HLA‐DR, in both NSTE‐ACS and post‐ACS patients. This is in line with previous findings of increased levels of circulating CD4^+^HLA‐DR^+^ T cells in both stable and acute conditions of CAD 21, 35.

Statins, a group of inhibitors of the 3‐hydroxy‐3‐methylglutaryl coenzyme A reductase, are potent cholesterol‐lowering agents; however, they may also exert anti‐inflammatory and immunomodulatory effects 36. Previous studies have shown that different statins, including simvastatin and atorvastatin, increase the numbers of CD4^+^CD25^+^FOXP3^+^ T cells in humans 37, 38. Almost all post‐ACS patients in our study population were treated with a statin (simvastatin or atorvastatin). We cannot exclude the possibility that statin treatment influenced the Tregs but, if so, this treatment apparently failed to restore Treg homeostasis.

The IL‐6 levels in plasma were similar in post‐ACS patients and control subjects. However, when PBMCs from post‐ACS patients were stimulated with LPS, they released IL‐6 and IL‐1β to a greater extent than PBMCs from control subjects, supporting the presence of a proinflammatory state in the patient group. Moreover, the *ex vivo* data indicated an inverse relationship between the LPS‐induced secretion of IL‐6 and IL‐1β and the functional capacity of Tregs, in particular nTregs. Although the results do not allow any conclusions about cause‐and‐effect relationships, they are in line with the finding from several experimental studies that Tregs not only inhibit effector T cells but also suppress the innate immune response 3, 4, 39. The capacity of human monocytes to produce IL‐6 in response to LPS was previously shown to be severely inhibited by Tregs 4.

To provide an estimate of atherosclerotic burden, noninvasive carotid ultrasound measurements were taken in post‐ACS patients and control subjects. The presence of plaques on both sides and also IMT on the right side were correlated with the levels of nTregs, but not with total Treg or mTreg levels. In two previous population‐based studies, no significant associations were found between total Tregs in blood and subclinical carotid atherosclerosis 10, 12. The present data suggest that it is instead the lack of nTregs within the Treg compartment that may have an impact on plaque development.

Our study has a number of limitations. Because it was a cross‐sectional study, we cannot draw any conclusions about changes in Treg status over time. It should also be noted that the clinical relevance of functional Treg assays is not known, as recently discussed by Sakaguchi *et al*. 2. We only included patients in the early phase of NSTE‐ACS, collecting blood samples before coronary intervention. The results may therefore not apply to patients with STE‐ACS. Ammirati *et al*. 21 demonstrated previously that Treg levels were significantly increased in STE‐ACS patients but reduced in NSTE‐ACS patients, compared with healthy control subjects or stable patients with CAD, thus highlighting potential differences in Treg behaviour between those with STE‐ACS and NSTE‐ACS.

In conclusion, this study is the first to show that Treg function and homeostasis are not preserved in patients with CAD, not only in the early phase of NSTE‐ACS but also 6–12 months after ACS. Further, our data suggest that both reduced thymic output and impaired capacity of Tregs to proliferate may underlie the Treg defect. Its physiological relevance may be reflected in the association with a proinflammatory cytokine profile and increased presence of carotid plaques, although we cannot exclude the possibility that the change in peripheral Tregs is merely a marker of disease. An intriguing question for future research is whether the failure to maintain Treg function and homeostasis in patients with CAD could provide a useful therapeutic target and, if so, whether interventions would be effective.

## Funding

This study was supported by grants from the Swedish Medical Research Council and the Swedish Heart‐Lung Foundation.

## Conflict of interest statement

No conflicts of interest to declare.
